# Brassicasterol with Dual Anti-Infective Properties against HSV-1 and *Mycobacterium tuberculosis*, and Cardiovascular Protective Effect: Nonclinical In Vitro and In Silico Assessments

**DOI:** 10.3390/biomedicines8050132

**Published:** 2020-05-24

**Authors:** Sherif T. S. Hassan

**Affiliations:** Department of Applied Ecology, Faculty of Environmental Sciences, Czech University of Life Sciences Prague, Kamýcká 129, 6-Suchdol, 16521 Prague, Czech Republic; sherif.hassan@seznam.cz; Tel.: +420-774-630-604

**Keywords:** brassicasterol, phytosterols, HSV, *Mycobacterium tuberculosis*, HSV-1 DNA polymerase, HSV-1 TK, human CDK2, ACE, UDP-galactopyranose mutase

## Abstract

While few studies have revealed the biological properties of brassicasterol, a phytosterol, against some biological and molecular targets, it is believed that there are still many activities yet to be studied. In this work, brassicasterol exerts a therapeutic utility in an in vitro setting against herpes simplex virus type 1 (HSV-1) and *Mycobacterium tuberculosis* (Mtb) as well as a considerable inhibitory property against human angiotensin-converting enzyme (ACE) that plays a dynamic role in regulating blood pressure. The antireplicative effect of brassicasterol against HSV-1 is remarkably detected (50% inhibitory concentration (IC_50_): 1.2 µM; selectivity index (SI): 41.7), while the potency of its effect is ameliorated through the combination with standard acyclovir with proper SI (IC_50_: 0.7 µM; SI: 71.4). Moreover, the capacity of this compound to induce an adequate level of antituberculosis activity against all Mtb strains examined (minimum inhibitory concentration values ranging from 1.9 to 2.4 µM) is revealed. The anti-ACE effect (12.3 µg/mL; 91.2% inhibition) is also ascertained. Molecular docking analyses propose that the mechanisms by which brassicasterol induces anti-HSV-1 and anti-Mtb might be related to inhibiting vital enzymes involved in HSV-1 replication and Mtb cell wall biosynthesis. In summary, the obtained results suggest that brassicasterol might be promising for future anti-HSV-1, antituberculosis, and anti-ACE drug design.

## 1. Introduction

Herpesviruses are one of the major causes of human viral diseases with highly infectious properties. Herpes simplex virus type 1 (HSV-1) is a type of alpha-herpesviruses with double-stranded DNA placed in an icosahedral capsid and a lipidic envelope made by various glycoproteins [1,2]. This pathogen is known to be a thoroughly contagious infection, which is frequent and endemic globally. Most HSV-1 infections are acquired during childhood, and infection is lifelong. Infections with HSV-1 are typically oral herpes but HSV-1 can also be transmitted to the genitals and causes genital herpes [3,4,5]. Oral herpes infection is commonly asymptomatic, however, in immunocompromised patients with advanced HIV infection, HSV-1 could generate obvious and severe symptoms with regular recurrences [6]. In some cases, complications such as encephalitis or keratitis were observed to be associated with HSV-1 infection [7]. Antiviral drugs, such as acyclovir, famciclovir, and valacyclovir are the best effective medicines to decrease the severity and frequency of HSV symptoms. These medications act as inhibitors of viral replication with reported undesirable effects. With the extensive use of these drugs in therapy, the problem of drug resistance has been established, leading to treatment failure [1,8].

Tuberculosis (TB) is an infectious disease induced by the Gram-negative bacterium *Mycobacterium tuberculosis* (Mtb) that most often infects the lungs and can also affect the brain, kidneys, or spine [9]. Patients infected with HIV, people with a weak immune system, and people with undernutrition are more likely to acquire active TB infection than healthy people [10]. TB infection is typically symptomatic, where the most frequent symptoms of active lung TB are cough with sputum and blood, at times, and fever, chest pains, weight loss, and overall weakness [11]. Although TB is a treatable disease, the extensive and or inappropriate use of anti-TB medications led to developing multidrug-resistant tuberculosis (MDR-TB), where drugs such as isoniazid and rifampicin, the two most potent first-line anti-TB medicines failed to treat the disease [12,13]. In 2018, the World Health Organization declared that MDR-TB continues to be a public health crisis and a health security threat with an increased level of resistance to first-line drugs [14].

Angiotensin-converting enzyme (ACE) is a key enzyme in the regulation of the renin-angiotensin system, with the ability to cleave angiotensin-I to angiotensin-II and hydrolyze several peptides [15]. It is known that angiotensin-II mainly circulates in the blood and triggers the muscles surrounding blood vessels to contract, thus narrowing the vessels, leading to an increase in blood pressure (hypertension) [16]. Therefore, ACE inhibition is an essential therapeutic approach in controlling acute and chronic hypertension, treating left ventricular dysfunction and heart failure, preventing strokes, and preventing and treating kidney disease (nephropathy), especially with patients suffering from hypertension or diabetes [17,18].

**Figure 1:**
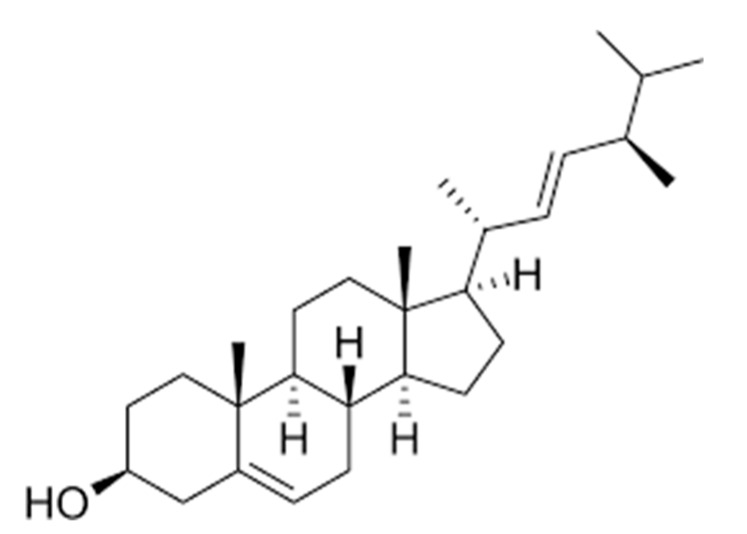
Chemical structure of brassicasterol.

Brassicasterol is a natural product that belongs to phytosterols (called plant sterol/stanol esters) and is biosynthesized by various unicellular algae and few terrestrial plants (Figure 1). This compound is a major sterol in rapeseed and canola oil and known to have nutritional value as a food additive [19]. Generally, cholesterol-lowering properties are the major beneficial effects of phytosterols on human health. However, the health benefits of phytosterols on humans have been a subject of debate for years [20]. Recently, a health claim on phytosterols has been clarified and verified by the U.S. Food and Drug Administration (FDA) with a statement: “Foods containing at least 0.65 g per serving of vegetable oil plant sterol esters, eaten twice a day with meals for a daily total intake of at least 1.3 g, as part of a diet low in saturated fat and cholesterol, may reduce the risk of heart disease.” [21].

So far, brassicasterol remains a little investigated phytosterol-type molecule with reported few biological activities [19,22,23]. Therefore, in this study, brassicasterol is examined using properly in vitro assay systems for its anti-infective properties against HSV-1 and Mtb along with its cardiovascular protective effect via inhibiting the activity of ACE. Additionally, molecular docking analyses are achieved to predict the mechanisms of action against the molecular targets as well as confirm the results obtained by the in vitro biological assay (for ACE). The investigated molecular targets for HSV-1 are HSV-1 DNA polymerase, HSV-1 thymidine kinase, and human cyclin-dependent kinase 2, while for Mtb it is UDP-galactopyranose mutase.

## 2. Materials and Methods

Compounds under investigation: Brassicasterol (purity ≥ 98%), standard acyclovir (ACV), and standard rifampicin (European Pharmacopoeia (EP) Reference Standard) were purchased from Sigma-Aldrich, Berlin, Germany, while standard captopril (EP reference standard) was acquired from Sigma Aldrich, Prague, Czech Republic. The combination of brassicasterol with ACV was prepared as a form of combinatory treatment.

### 2.1. Antiherpetic Activity

#### 2.1.1. Preparation of Vero Cells and the Virus

Vero cells (ATCC: CCL 81^TM^; UK) and an ACV- susceptible strain of HSV-1 (KOS) were collected from Motol University Hospital (MUH; Prague, Czech Republic). Vero cells were prepared and grown following the previously reported protocol [2,6]. HSV-1 stocks were obtained after propagation of the virus in Vero cells. A plaque assay was further used to titrate the HSV-1 stocks on the basis of the plaque-forming unit (PFU) and the HSV-1 stocks were then kept at –80 °C, as previously specified [2,6].

#### 2.1.2. Cell Viability Assay for Determination of Cytotoxicity

The viability of Vero cells was assessed by the neutral red dye-uptake assay, as previously detailed [8]. Briefly, the experiment was initiated by treating Vero cell monolayers grown in 96-well microtiter plates with two-fold serial dilutions of the test compounds (100 µM as a starting concentration) and then exposed to incubation for 48 h at 37 °C in 5% CO_2_. After incubation, the treated cells were examined for the morphological changes by an inverted optical microscope (Leitz, Wetzlar, Germany) and the maximum non-toxic concentrations (MNTC) were ascertained. The 50% cytotoxic concentrations (CC_50_; expressed in µM) of test compounds were calculated as the concentrations that decreased the cell viability by 50% when compared to the untreated control cells.

#### 2.1.3. Plaque Reduction Assay for Determination of Antiherpetic Activity

To evaluate the antiviral activity against the replication of HSV-1, a plaque reduction assay was performed using ACV as a standard antiherpetic drug as earlier detailed [2,6]. The assay was initiated by infecting Vero cell monolayers with the virus (100 PFU) for 1 h at 37 °C. The infected cells were further overlapped with Eagle’s minimum essential medium containing carboxymethyl cellulose (1.5%; Sigma-Aldrich, Berlin, Germany) and treated with test compounds at different concentrations. The untreated cells were considered as controls. To determine the plaque reduction, cells were incubated for 72 h at 37 °C and then dyed with naphthol blue-black (Sigma-Aldrich, Berlin, Germany). The concentrations of test molecules that diminished 50% of the number of HSV-1 plaques were assessed in comparison with the untreated control cells and expressed as 50% inhibitory concentration (IC_50_ in µM) values. The ratio CC_50_/IC_50_ was assigned for each compound to calculate the selectivity index (SI).

### 2.2. Antimycobacterial Assay

#### 2.2.1. Bacterial Strains, Identification protocols, and Culturing Procedures

The anti-Mtb activity was processed using an in vitro microdilution assay with ten Mtb strains (CI1–CI10; bacterial isolates acquired from MUH, Prague, Czech Republic), along with a reference strain (H37Rv; CNCTC My 331-88: ATCC 27294; Czech National Collection of Type Cultures (CNCTC), National Institute of Public Health, Prague, Czech Republic. For identification, all Mtb isolates were subjected to various microbiological, biochemical, and molecular protocols following the accredited guideline of Clinical and Laboratory Standards Institute (CLSI) [24]. All bacterial strains used in this study were identified as rifampicin-susceptible and were cultured and grown as previously described by CLSI guideline [25].

#### 2.2.2. Drug Susceptibility Assay

Minimum inhibitory concentrations (MICs) of test substances (brassicasterol and rifampicin) against mycobacterial strains were investigated by employing a 96-well plate microdilution broth method under optimal assay conditions according to the specified procedure of the CLSI guideline with slight modification [25]. Briefly, the broth Middlebrook OADC (Oleic Albumin Dextrose Catalase) growth supplement was used as a supplement in 7H9 liquid medium to culture Mtb (Sigma-Aldrich, Berlin, Germany) at pH (6.6). Rifampicin was applied as a positive control, while Dimethyl sulfoxide (DMSO; 1%) and the broth were utilized as the negative controls. The test drugs were prepared in DMSO with broth (25 µL of DMSO solution in 4 mL of broth) and an aliquot of 100 µL was placed into microplate wells. An isotonic saline solution was used to suspend Mtb inocula with an adjusted density of 0.5–1.0 McFarland. Further, the suspensions (diluted by 10^−1^) were employed to inoculate the testing wells with a mixture of suspension (100 µL) and the test drugs (100 µL). After five days of incubation, an aliquot of 30 µL of Alamar Blue solution containing a mixture (1:1) of an aqueous solution of resazurin sodium salt (0.1%) and Tween 80 (10%) was supplemented. Further, the results were finalized after 24 h of incubation and expressed as MIC values that hindered the blue to pink color change.

### 2.3. Anti-Angiotensin-Converting Enzyme (ACE) Assay

Brassicasterol and standard captopril were investigated for their anti-ACE properties. ACE inhibition activities of test compounds were determined by a spectrophotometric method based on the production of hippuric acid from hippuryl-_L_-histidyl-_L_-leucine (HHL; substrate) [15]. Concisely, the assay was performed by incubating the test substances at a fixed concentration of 12.3 µg/mL with 120 mU/mL of human ACE (Sigma-Aldrich, Prague, Czech Republic) prepared in Tris Buffer (50 mM, pH = 8.3) for 80 min at 37 °C. After incubation, ACE activity was initiated by adding 110 μL of HHL (10 mM; Sigma-Aldrich, Prague, Czech Republic) to the mixture. A spectrophotometer (UV–VIS; SPECTROstar^Nano^ BMG Labtech, Ortenberg, Germany) was utilized to detect the hippuric acid from the reaction at λ 228 nm and, subsequently, the degree of inhibition (%) was calculated.

### 2.4. In Silico Molecular Docking Analyses

The molecular docking studies were performed following the previously described protocol [26] and processed with a PyRx virtual screening tool merged with Autodock VINA software (version 0.8, The Scripps Research Institute, La Jolla, CA, USA). Discovery studio visualizer version v19.1.0.18287 (BIOVIA, San Diego, CA, USA) was further employed to graphically illustrate the docking results.

The three-dimensional (3D) crystal structure of HSV-1 DNA polymerase (PDB ID: 2GV9), the 3D-crystal structure of thymidine kinase from HSV-1 complexed with 5-iododeoxyuridine (HSV-1 TK; PDB ID: 1KI7), the 3D-crystal structure of human cyclin-dependent kinase 2 in complex with roscovitine (CDK2; PDB ID: 2A4L), the 3D-crystal structure of UDP-galactopyranose mutase from Mtb docked with UDP (UGM; PDB ID: 4RPJ), and the 3D-crystal structure of human angiotensin-converting enzyme complexed with captopril (ACE; PDB ID: 1UZF) were obtained from the RCSB Protein Data Bank (www.rcsb.org), while the SDF file of the 3D-structure of brassicasterol (CID: 5281327) was retrieved from PubChem database.

To validate the molecular docking outcomes, 5-iododeoxyuridine, roscovitine, UDP, and captopril were removed from (PDB ID: 1KI7), (PDB ID: 2A4L), (PDB ID: 4RPJ), and (PDB ID: 1UZF), respectively and re-docked back into their receptors. The docking results were expressed as binding energy values (kcal/mol) of ligand-receptor complexes; these are based on hydrogen bond, hydrophobic, and electrostatic interactions. All required docking settings including the preparation of PDBQT files for the receptors and ligands, determination of binding sites, the protonation state, calculations, and the overall charges were established as hitherto described [26].

## 3. Results and Discussion

### 3.1. Assessment of Cytotoxicity and Antiherpetic Properties

As shown in Table 1, the cytotoxicity (evaluated by the neutral red dye-uptake assay using Vero cells and expressed as 50% cytotoxic concentration (CC_50_) value) for brassicasterol, brassicasterol combined with acyclovir (ACV), along with standard ACV was detected to be greater than 50 µM. After the determination of cytotoxicity, Vero cells were infected with HSV-1. Further, the infected cells were treated with the test compounds using an in vitro plaque reduction assay.

All test molecules showed obvious antiviral properties against the replication of HSV-1 (expressed as 50% inhibitory concentration (IC_50_) value), where the treatment with brassicasterol demonstrated greater anti-HSV-1 activity (IC_50_: 1.2 μM; selectivity index (SI): 41.7) than ACV (IC_50_: 2.1 μM; SI: 23.8). Additionally, the potency of anti-HSV-1 activity was improved through the combinatory treatment of brassicasterol with ACV (IC_50_: 0.7 µM; SI: 71.4) compared with that of ACV as a single treatment.

The effective treatment of HSV remains the greatest global challenge in medicine, where the current pharmacological treatment is complicated by the rapid development of drug resistance. Therefore, and to combat and eliminate drug resistance to antiherpetic drugs, new efficient and safe medicines with the ability to provide less resistance are urgently required to enter the clinical practice [1,15].

The acquired results confirmed that brassicasterol has a promising anti-HSV-1 property via inhibiting the viral replication, and its cellular toxicity on Vero cells was observed at a concentration (>50 µM) higher than its IC_50_ value, which means that this compound has an acceptable level of selectivity (expressed as SI) towards the target virus. It has been previously claimed that combinatory treatment with clinically antiherpetic drugs is a valuable option for improving the treatment efficacy of HSV diseases [27]. Hence, the obtained results support this claim by showing the capacity of brassicasterol to increase the anti-HSV activity of standard ACV via a combinatory treatment.

To date, no studies have claimed the antiherpetic properties of brassicasterol. Therefore, the obtained results by this research could be rationalized with other studies in which various natural products have shown in various preclinical investigations the capability of inducing excellent anti-HSV activities with diverse mechanisms of action that could break barriers to novel anti-HSV drug development. This has been documented in a recent review article in which a large number of natural biomolecules extracted from various natural sources with outstanding activities against HSV infections were comprehensively reviewed [28].

### 3.2. Assessment of Antituberculosis Activity

Brassicasterol was examined for antituberculosis activity by microdilution susceptibility assay. The in vitro susceptibility test was processed with ten clinical isolates of Mtb along with a standard strain. Brassicasterol displayed an adequate level of inhibitory effects on the growth of all Mtb strains tested with MIC values ranging from 1.9 to 2.4 µM (Table 2), while the MIC values for the standard antitubercular drug rifampicin were also recorded.

Although substantial advancements have been achieved during the past few decades in the diagnosis and treatment of TB, the problem of antibiotic resistance remains a challenge [29]. In the present study, brassicasterol showed apparent antitubercular properties via inhibiting the growth of all Mtb strains tested, while no bactericidal effect was observed. In further investigation, and to improve the anti-Mtb activity of brassicasterol, combinatory treatment with standard drug rifampicin was performed. Unfortunately, no positive outcomes were detected to reveal any synergy, additive, or even antagonistic effects.

While several phytosterols derived from marine algae and plants were observed with a slight to moderate degree of antimicrobial actions on Mtb [30,31,32,33], their mechanisms of action remain poorly examined. This could be related to their low potency as antitubercular agents, which in turn limits performing further investigations to unveil the mechanisms of action. To the best of the author’s knowledge, this research reports the first finding on the anti-Mtb effect of brassicasterol.

### 3.3. Evaluation of Anti-Angiotensin-Converting Enzyme Activity

The inhibition of human ACE activity by brassicasterol was measured by the release of hippuric acid from the enzyme reaction. The activity of ACE was markedly diminished by brassicasterol at a concentration of 12.3 µg/mL with 91.2% inhibition compared with that of standard captopril (99.1% at a concentration of 12.3 µg/mL) (Table 3).

ACE inhibitors are known to decrease the production of angiotensin-II, leading to lowering blood pressure, and hence are essential for curing hypertension, heart failure, and other cardiovascular and renal diseases [15]. The re-emerging interest in finding new ACE inhibitors has become urgent, due to the occurrence of undesirable side effects associated with treatment with clinically ACE inhibitors such as captopril, lisinopril, and benazepril [34]. Phytosterols have previously been claimed for their ability to control ACE levels without direct inhibition of ACE. For instance, fucosterol was observed to reduce the ACE levels in endothelial cells by impeding the synthesis of glucocorticoid receptors involved in the regulation of enzyme levels [35]. On the other hand, the current study documents the first investigation on brassicasterol as a direct inhibitor of ACE activity.

### 3.4. Molecular Docking Evaluation

#### 3.4.1. Brassicasterol with Enzymes Involved in Viral Replication

**Figure 2:**
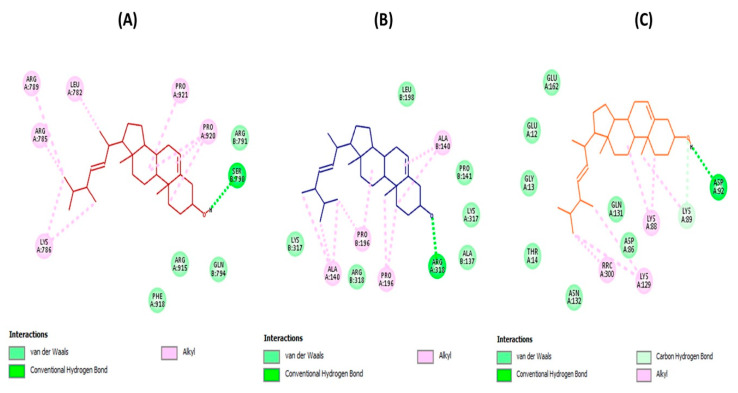
Molecular docking analyses show the molecular interactions and the binding modes of brassicasterol with the active sites of HSV-1 DNA polymerase (**A**), HSV-1 thymidine kinase (**B**), and human cyclin-dependent kinase 2 (**C**), where interactions (hydrogen bond, carbon-hydrogen bond, alkyl hydrophobic, and van der Waals interactions) are presented in a two-dimensional model. Interacting amino acid residues of enzymes active sites with the key functional groups of brassicasterol are shown.

As depicted in Figure 2, the docking results revealed the substantial ability of brassicasterol to suitably bind to the active sites of HSV-1 DNA polymerase, HSV-1 thymidine kinase (HSV-1 TK) and human cyclin-dependent kinase 2 (CDK2) with detected binding energy values –8.0, –3.3, and –6.5 kcal/mol, respectively. The molecular interactions have been established by forming several fundamental interactions with functional groups of brassicasterol and the interacting amino acid residues of the active sites of target enzymes. These interactions were observed to be hydrogen bond, carbon-hydrogen bond, and hydrophobic (alkyl hydrophobic) along with van der Waals contacts.

Targeting viral and cellular enzymes that perform significant functions during the HSV-1 replication cycle could help the development of effective broad-spectrum antiherpetic drugs. For instance, HSV-1 DNA polymerase was reported to be an essential enzyme required for viral replication [3], while HSV-1 TK is an important enzyme that catalyzes the transfer of the gamma-phospho group of ATP to thymidine to generate dTMP in the salvage pathway of pyrimidine synthesis, and hence the dTMP serves as a substrate for DNA polymerase during viral DNA replication [36].

It has also been reported that cellular proteins are potential targets for antiviral therapy since most viruses depend on specific cellular proteins for replication. Particularly, CDK2 has been described as a potential target for designing novel anti-HSV-1 drugs [37,38]. According to the currently available evidence, CDK2 was observed to be involved in viral replication and its inhibitors displayed notable anti-HSV-1 activities [39,40,41]. An additional study unveiled that HSV-1 reactivated in neurons expressing CDK2, and the level of this protein is implicated in the reactivation process [42]. Given together the available data, CDK2 was therefore selected as a target for performing the molecular docking investigations. On the other hand, additional in vitro and in silico studies are necessary to be performed against all CDK families to verify the selectivity of brassicasterol against CDK2.

Unlike the critical role in the viral infection cycle, CDK2 belongs to protein kinases that play a vital role in the eukaryotic cell division cycle. Inhibition of this protein poses a central role in DNA damage-induced cell cycle arrest and DNA repair; however, some negative effects could also be generated [43]. CDK2 is one of the most significant molecular targets identified for cancer-drug discovery and inhibitors of this protein are considered promising chemotherapeutics to combat various types of cancer [44].

#### 3.4.2. Brassicasterol with UDP-Galactopyranose Mutase

**Figure 3:**
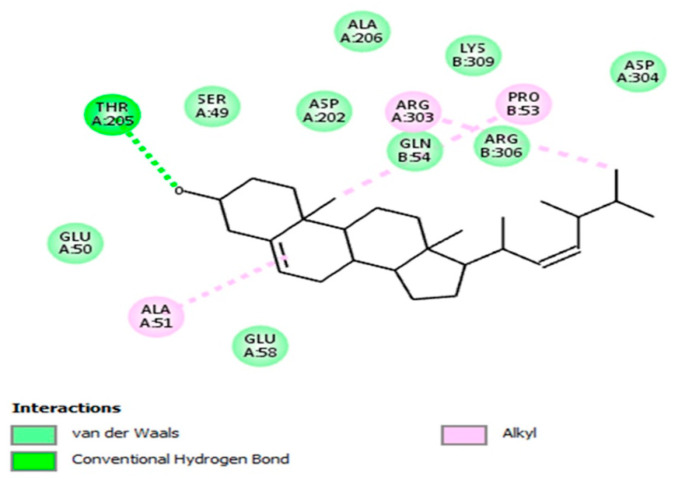
Molecular docking analysis reveals the molecular interaction and the binding mode of brassicasterol with the active site of UDP-galactopyranose mutase (UGM; from *Mycobacterium tuberculosis*), where interactions (hydrogen bond, alkyl hydrophobic, and van der Waals interactions) are shown in a two-dimensional model. Interacting amino acid residues of UGM active site with the key functional groups of brassicasterol are displayed.

UDP-galactopyranose mutase (UGM) is a critical enzyme implicated in the metabolism of galactofuranose, which is known to be a key component of the Mtb cell wall [45]. The docking result of the brassicasterol-UGM complex revealed notable binding mode (Figure 3) with binding energy (–8.1 kcal/mol). The molecular interaction was revealed, where the functional groups of brassicasterol have formed with the interacting amino acid residues of UGM active site several crucial interactions such as hydrogen bond, hydrophobic (alkyl hydrophobic), and van der Waals interactions.

Inhibition of mycobacterial cell wall biosynthesis is one of the most effective mechanisms of action of various antibiotics. Mycobacterial cell wall contains a variety of enzymes that play a significant role in its biosynthesis. Therefore, targeting these enzymes is a striking approach for designing effective anti-Mtb drugs [46].

#### 3.4.3. Brassicasterol with Angiotensin-Converting Enzyme

**Figure 4:**
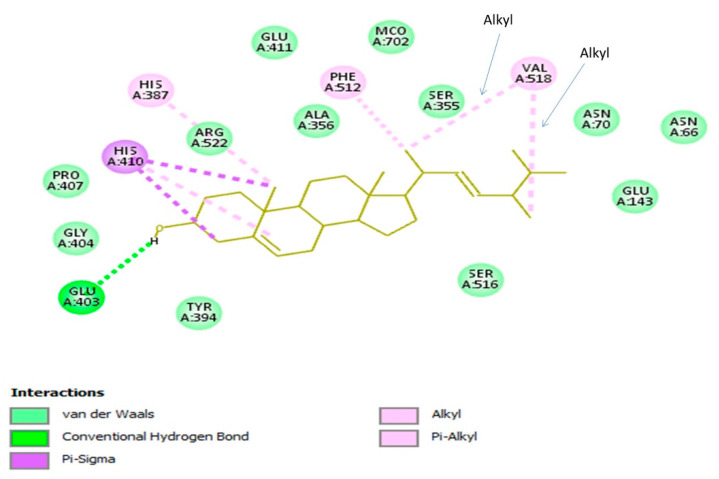
Molecular docking analysis discloses the molecular interaction and the binding mode of brassicasterol with the active site of human angiotensin-converting enzyme (ACE), where interactions (hydrogen bond, alkyl, Pi-alkyl, and Pi-sigma hydrophobic, and van der Waals interactions) are displayed in a two-dimensional model. Interacting amino acid residues of ACE active site with the key functional groups of brassicasterol are shown.

The molecular interaction of brassicasterol with the active site of the angiotensin-converting enzyme (ACE) was explored (Figure 4). The brassicasterol-ACE complex was created with a binding energy value of –9.9 kcal/mol by establishing important hydrogen bond, hydrophobic, and van der Waals interactions. It has been ascertained that zinc is important to the catalytic action of ACE [47], however, brassicasterol was observed to bind to different active pockets than the zinc active-site. Such an outcome may indicate that this compound has a specific type of inhibition that needs to be disclosed in further in vitro investigation.

## 4. Conclusions

In addition to the nutritional and biological values induced by brassicasterol, the outcome of this work has drawn attention to the novel biological properties of this compound, in which anti-infective actions against HSV-1 and Mtb along with cardiovascular protective effects (through inhibition of ACE activity) were ascertained by in vitro and in silico molecular docking studies. Although brassicasterol showed in vitro promising therapeutic values against the investigated targets, further in-depth in vivo investigations are required to validate the obtained results and to unveil the mechanisms of action along with integrated pharmacokinetic and pharmacodynamic studies. Moreover, additional in vitro studies are also necessary to confirm the findings achieved by molecular docking studies.

## Figures and Tables

**Table 1:** Antireplicative actions of brassicasterol and brassicasterol in combination with acyclovir against herpes simplex virus type 1 compared to standard acyclovir along with cytotoxicity properties.

Molecules	CC_50_ (μM)	IC_50_ (μM)	SI (CC_50_/IC_50_)
Brassicasterol	>50	1.2 ± 0.12	>41.7
Brassicasterol combined with ACV	>50	0.7 ± 0.24	>71.4
ACV (standard)	>50	2.1 ± 0.13	>23.8

The acquired values are means ± standard deviation (SD) of three independent measurements assayed in duplicate. CC_50_: 50% cytotoxic concentration, IC_50_: 50% inhibitory concentration. CC_50_ and IC_50_ values were determined by nonlinear regressions of concentration-response curves. The differences between treatments with test molecules and the controls were analyzed using a one-way ANOVA test tracked by post-hoc comparison tests (Dunnett and Student–Newman–Kuels), where statistical significance was *p* < 0.05, SI: Selectivity index defined as the ratio CC_50_/IC_50_, ACV: Acyclovir. For performing the statistical analyses, PRISM software version 8.0 (GraphPad Software, Inc., La Jolla, CA, USA) was employed.

**Table 2:** Antituberculosis activity (MIC, µM) of test compounds.

Mycobacterial Strains	MIC (µM)	
	Brassicasterol	Rifampicin
Mtb ^a^	1.9 ± 0.12	0.1 ± 0.01
Mtb-CI1 ^b^	2.0 ± 0.13	0.2 ± 0.02
Mtb-CI2 ^b^	2.4 ± 0.15	0.2 ± 0.03
Mtb-CI3 ^b^	2.2 ± 0.14	0.3 ± 0.02
Mtb-CI4 ^b^	2.1 ± 0.14	0.3 ± 0.02
Mtb-CI5 ^b^	1.9 ± 0.13	0.1 ± 0.02
Mtb-CI6 ^b^	1.9 ± 0.13	0.3 ± 0.01
Mtb-CI7 ^b^	2.1 ± 0.14	0.2 ± 0.01
Mtb-CI8 ^b^	1.9 ± 0.11	0.4 ± 0.03
Mtb-CI9 ^b^	2.1 ± 0.13	0.3 ± 0.02
Mtb-CI10 ^b^	2.0 ± 0.15	0.1 ± 0.01

All recorded values are means ± standard deviation (SD) of three independent experiments assayed in triplicate. ^a^ Mtb: *Mycobacterium tuberculosis* (reference strain; H37Rv CNCTC My 331-88/ATCC 27294); ^b^ Mtb: *Mycobacterium tuberculosis* clinical strains; MIC: minimum inhibitory concentration. All presented data were processed by PRISM software (GraphPad Software, Inc., La Jolla, CA, USA; version 8.0).

**Table 3:** Inactivation properties of test compounds against the human angiotensin-converting enzyme.

Compounds	% Inhibition
Brassicasterol	91.2 ± 0.43
Captopril (standard)	99.1 ± 0.12
ACE-catalyzed reaction (no inhibition)	-

All exhibited values are the mean ± standard deviation (SD) (*n* = 3). ACE: Angiotensin-converting enzyme. The presented data were processed by PRISM software version 8.0 (GraphPad Software, Inc., La Jolla, CA, USA).
